# Estimated Cost-effectiveness of Newborn Screening for Congenital Cytomegalovirus Infection in China Using a Markov Model

**DOI:** 10.1001/jamanetworkopen.2020.23949

**Published:** 2020-12-04

**Authors:** Kai Chen, Yaqin Zhong, Yuanyuan Gu, Rajan Sharma, Muting Li, Jinjun Zhou, Youjia Wu, Yuexia Gao, Gang Qin

**Affiliations:** 1 Department of Internal Medicine, Nantong University Medical School, Nantong, Jiangsu, China; 2 Department of Health Management, Nantong University School of Public Health, Nantong, Jiangsu, China; 3 Centre for the Health Economy, Macquarie University, Sydney, New South Wales, Australia; 4 Department of Pediatrics, Nantong Maternal and Child Health Hospital, Nantong University, Nantong, Jiangsu, China; 5 Department of Pediatrics, Nantong University Affiliated Hospital, Nantong, Jiangsu, China; 6 Department of Infectious Diseases, Nantong Third People’s Hospital Affiliated to Nantong University, Nantong, Jiangsu, China

## Abstract

**Importance:**

Congenital cytomegalovirus infection (cCMVi) is one of the most common infections associated with childhood hearing loss. Prevention and mitigation of cCMVi-related hearing loss will require an increase in newborn screening, which is not yet available in China.

**Objective:**

To estimate the cost-effectiveness of newborn screening strategies for cCMVi from the perspective of the Chinese health care system.

**Design, Setting, and Participants:**

A decision tree for a simulated cohort population of 15 000 000 live births was developed to compare the costs and health effects of 3 mutually exclusive interventions: (1) no screening, (2) targeted screening using CMV polymerase chain reaction assay for newborns who fail a universal hearing screening, and (3) universal screening for CMV among all newborns. Markov diagrams were used to evaluate the lifetime horizon (76 years).

**Main Outcomes and Measures:**

Cost, hearing-related health outcomes, and incremental cost-effectiveness ratios (ICERs) were estimated based on a direct medical costs perspective. Costs and ICERs were reported in 2018 US dollars.

**Results:**

Incidence of cCMVi among newborns was reported to be approximately 0.7% in China. Targeted screening was less costly but also less effective than universal screening, identifying 41% of cases needing antiviral treatment and preventing nearly half of less severe or profound hearing loss. To avoid 1 CMV-related severe or profound hearing loss, 13 and 16 newborns need to be treated by targeted and universal screening, respectively. The ICERs of targeted and universal screening vs no screening were $79 and $2087 per quality-adjusted life-year gained, respectively, at the discounted rate of 3.5%. Both screening options were cost-effective for the Chinese health care system based on the willingness-to-pay threshold of 3 × gross domestic product per capita. The sensitivity analysis showed that the prevalence of cCMVi, as well as diagnosis and treatment costs, were key factors that may be associated with decision-making.

**Conclusions and Relevance:**

To achieve cost-effectiveness and best health outcomes, universal screening could be considered for the Chinese population. While the results are specific to China, the model may easily be adapted for other countries.

## Key Points

**Question:**

What strategies are most cost-effective for preventing and mitigating congenital cytomegalovirus infection (cCMVi) and associated hearing loss in China?

**Findings:**

In this modeling study, targeted and universal newborn cCMVi screening was associated with a reduction in the number of cases of childhood hearing loss by 820 (597-1193) and 2316 (1655-3308) each year, respectively. The incremental cost-effectiveness ratios of targeted and universal screening vs no screening were $79 and $2087 per quality-adjusted life-year gained, respectively, at the discounted rate of 3.5%.

**Meaning:**

The findings suggest that universal cCMVi screening could be considered a cost-effective extension of existing newborn screening programs in the setting of the Chinese health care system.

## Teaser

This modeling study examines the cost-effectiveness of newborn screening strategies for congential cytomegalovirus infection from the perspective of the Chinese health care system.

## Introduction

Congenital cytomegalovirus infection (cCMVi) is one of the most frequently occurring but underrecognized global health problems. Globally, around 2 to 20 per 1000 newborns develop cCMVi of all newborns.^1^ The economic effect of cCMVi was estimated to be $1.9 billion per year in the US by the Institute of Medicine 2 decades ago.^2^ Maternal primary and nonprimary infection (reinfection or reactivation) of CMV during pregnancy can result in mother-to-child transmission. At present, treatment with specific immunoglobulin in pregnant women with primary CMV infection to prevent mother-to-child transmission is not recommended, as, to our knowledge, studies have not yet conclusively shown a benefit.^3,4^ Around 50% to 70% and more than 70% of women who could become pregnant are seropositive in high-income countries (eg, US, UK, Australia, and Japan) and middle-income countries (eg, China, India, Brazil, and South Africa), respectively.^1^ Newborns with cCMVi can be categorized as symptomatic (approximately 14%) or asymptomatic based on clinical symptoms and signs.^5^ Sensorineural hearing loss (SNHL) is the most common sequela, affecting 34% to 41% of symptomatic newborns. Meanwhile, hearing loss will have a late-onset or progressive course in 7% to 11% of those asymptomatic infants with infection.^6^ At the critical stage of development and learning, early childhood hearing loss may result in delayed psychosocial development and communication, affecting long-term educational and employment opportunities.^7^

The diagnosis of cCMVi can be made using saliva or urine within the first 3 weeks of life by the polymerase chain reaction (PCR) analysis.^8,9^ Early detection provides an opportunity for antiviral treatment within the first month.^10^ Early identification and intervention may result in improved short-term and long-term outcomes. This could be attributed to timely antiviral treatment, earlier follow-up, and rehabilitation services. Thus, several health systems across the world are considering using newborn screening programs to test every newborn for cCMVi. It has already been used as an effective way to detect all infants at risk of CMV sequelae in Japan.^11^ Meanwhile, the targeted screening has been used by some health systems as an alternative to the universal screening.^12,13,14,15^ It was hailed as an important intervention milestone in the US,^16^ but the results from 7 US medical centers suggest that targeted screening could detect only around 40% of infants with CMV-related SNHL.^17^ It may also not be able to identify infants with cCMVi who are at risk of late-onset SNHL. Additionally, targeted screening has been found to have a low yield.^18^

Cytomegalovirus seroprevalence among pregnant women and infection incidence among newborns were reported to be 96.2% and 0.7%, respectively, in China.^19^ Until now, little has been done to stop CMV from affecting newborns in China. A growing number of medical professionals and CMV experts have appealed for public policy and legislation mandating newborn CMV screening.^20^ However, there is a dilemma regarding whether universal or targeted screening should be used to identify children with cCMVi.^11,16^ Results from economic evaluations in other countries may not apply in the Chinese context, given the substantial difference in health systems. In this study, we conducted what is, to our knowledge, the first economic evaluation that compares the cost-effectiveness of universal and targeted cCMVi screening strategies with no screening in China. Understanding the costs and value from screening program use may be valuable in terms of a future scale-up in prevention efforts.

## Methods

### Data Sources

Most of the model parameters associated with cost, effectiveness, and status transition were carefully selected from previous studies. Some of the model parameters were directly quoted and others were calculated based on the detailed data. The methods and reporting of this evaluation were consistent with the Consolidated Health Economic Evaluation Reporting StandardsConsolidated Health Economic Evaluation Reporting Standards (CHEERS) reporting guideline checklist.^21^ This study was deemed exempt from ethical review and the need for informed consent by the Nantong Third People's Hospital institutional review board because it is a simulation-based study and not human participants research.

### Model Structure

**Figure 1.**
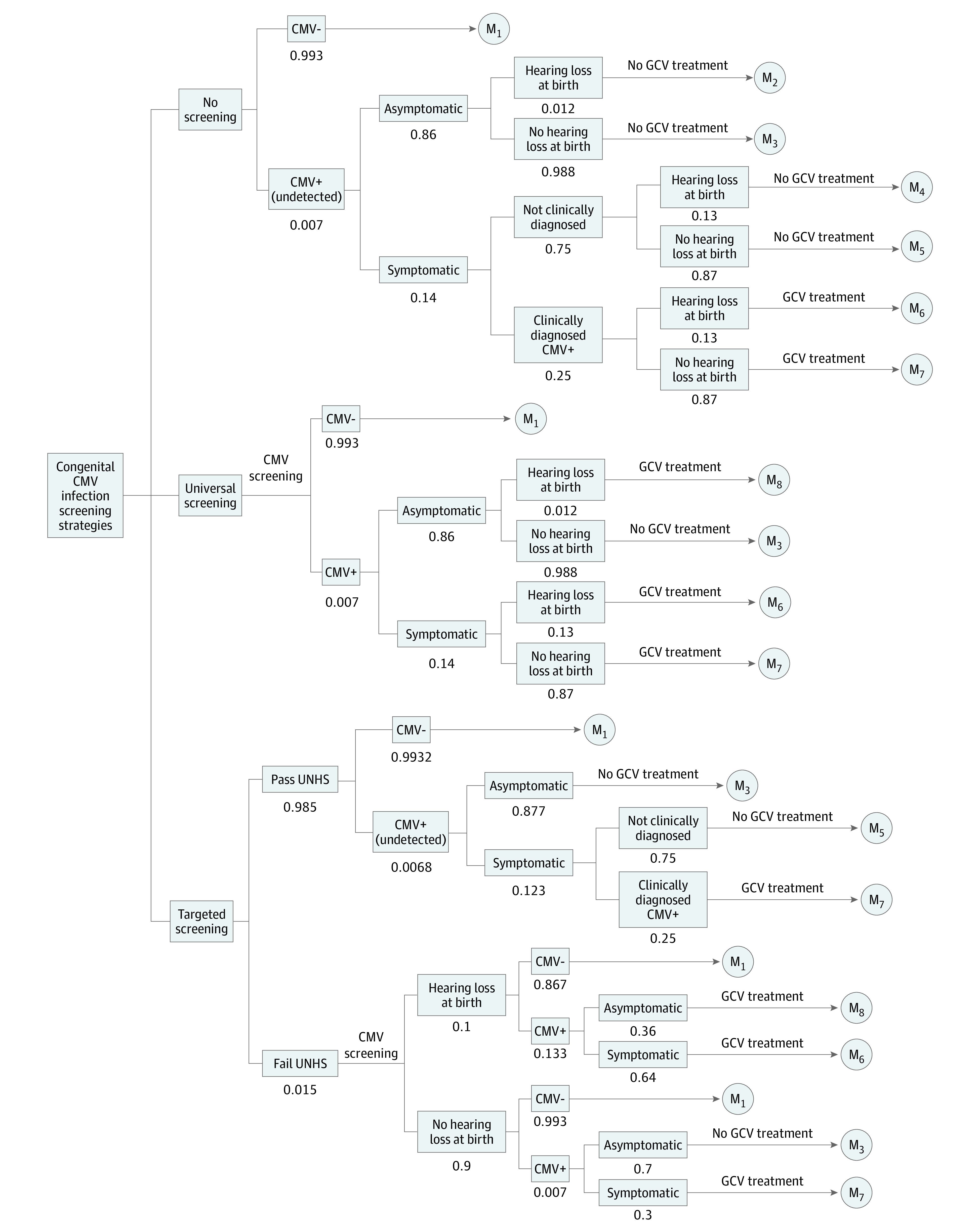
Decision Tree Structure of 3 Congenital Cytomegalovirus (CMV) Infection Screening Strategies GCV indicates ganciclovir; M, Markov model; UNHS, universal newborn hearing screening.

We built a decision-analytic Markov model using TreeAge Pro 2019 (TreeAge Inc). This model consisted of a decision tree (Figure 1) reflecting the 3 simulated strategies and the proportion of children with a diagnosis followed by Markov models reflecting the subsequent progression or remission of hearing loss over lifetime. Strategy 1 was no screening and was based on the current status. It was assumed that 25% of all symptomatic cases would have received a clinical diagnosis without the screening.^5,22^ Strategy 2 was targeted screening or hearing-targeted screening. Only those infants with failed universal newborn hearing screening underwent testing for CMV infection. We assumed that 1.5% of all infants have failed a hearing screening.^5^ Strategy 3 was universal screening. Under this strategy, all infants underwent testing for CMV infection using a PCR analysis of saliva and/or urine after birth.

An economic model was parameterized with 15 million children (the number of live births in China in 2018).^23^ In the decision tree, all children were modeled in each of the 3 cCMVi screening strategy arms. The CMV diagnostic evaluation was assumed to be 100% accurate.^8,9^ All newborns with cCMVi who had symptoms and/or hearing loss would receive antiviral treatment with intravenous ganciclovir (GCV), which was initiated within the first month of life.^20^ Children from the decision tree subsequently enter the first cycle of the Markov models. The natural history of CMV-related hearing loss and baseline incidence were based on a literature review.^5,19,24,25,26,27^

The Markov models for children with hearing loss at birth consisted of 6 health states (eFigure 1 in the Supplement): (1) normal hearing, (2) mild to moderate (M/M) hearing loss, (3) severe to profound (S/P) hearing loss, (4) cochlear implant, (5) no cochlear implant, and (6) death. The Markov models for children with normal hearing at birth consisted of 6 health states (eFigure 1 in the Supplement): (1) no hearing loss, (2) late-onset M/M hearing loss, (3) late-onset S/P hearing loss, (4) cochlear implant, (5) no cochlear implant, and (6) death. Mild to moderate hearing loss and S/P hearing loss are defined as hearing loss of 26 to 60 dB HL and 61 or more dB HL, respectively. Severe to profound hearing loss is the main indication of cochlear implant.^5,28^

China’s basic medical insurance package currently does not cover costs associated with cochlear implant. We assumed that 50% of children with S/P hearing loss received a cochlear implant based on expert consultation and literature review.^5,14^ Based on several cochlear implant rehabilitation projects and future trends, we estimated economic parameters according to different proportions of cochlear implant, ranging from 20% to 100% in the sensitivity analysis. Age-specific overall annual mortality rates for the general population were obtained from the national bureau of statistics.^29^

### Model Input

The inputs considered in the model are presented in Table 1.^5,10,19,24,25,26,27,28,30,31,32,33,34,35^ Prevalence data of cCMVi were obtained from reports published by Chinese and international professional groups.^10,19^ The transitional probabilities to reflect progression and remission among different severity levels were estimated based on previous studies.^5,24,25,26,27,30,31,32,33^ For studies in which multiyears rather than 1-year incidence was reported, the formula *r* = −log(1 − *p*)/*t* was used to calculate the 1-year incidence, with *r* representing the 1-year incidence and *p* denoting the cumulative incidence over the length of interval *t*.^36^ As late-onset hearing loss occurs mostly in childhood, especially before age 6 years,^22,37^ we assumed that late-onset hearing loss only occurred before age 6 years in the model. All direct medical costs were measured in Chinese yuan and were converted to US dollars at an exchange rate of 6.67 yuan per dollar. Cytomegalovirus screening was estimated to cost $15 per newborn PCR test based on the data from Peking University First Hospital. The treatment course of intravenous ganciclovir (GCV) was 6 weeks in the base-case analysis.^20^ The medical costs (including drugs, intravenous consumables, care costs, bed fees, and follow-up tests, including hepatic, kidney function, and routine blood tests) were estimated to be $675. We followed the international consensus recommendations^10^ and estimated the cost of the treatment of oral valganciclovir (VGCV). The cost of antiviral therapy was estimated to be between $600 and $1350. We also estimated the cost of the first year with cochlear implant, the subsequent annual costs, the cost of a hearing check, and the annual cost of M/M and S/P hearing loss. These costs were estimated based on a cost-effectiveness analysis on pediatric cochlear implant in China.^28^

**Table 1.** Baseline Values, Range, and Reference of Model Parameters

Input	Value (range)	Source
Prevalence of congenital CMV infection	0.007 (0.002-0.020)	Rawlinson et al,2017^10^; Wang et al, 2017^19^
**Transition probabilities (annual)**		
Symptomatic newborns with hearing loss at birth		
Treatment		
Improvement of hearing loss	0.188 (±20%)^a^	Mazzaferri et al, 2017^30^
Progress of hearing loss	0.011 (±20%)^a^	Bilavsky et al, 2016^31^
No treatment		
Improvement of hearing loss	0.022 (±20%)	Royackers et al, 2011^24^
Progress of hearing loss	0.075 (±20%)	Royackers et al, 2011^24^
Symptomatic newborns without hearing loss at birth		
Late-onset hearing loss		
Treatment		
M/M	0.001 (±20%)^a^	Ohyama et al, 2019^32^; Bilavsky et al, 2016^31^
S/P	0.0004 (±20%)^a^	Ohyama et al, 2019^32^; Bilavsky et al, 2016^31^
No treatment		
M/M	0.021 (±20%)	Gantt et al, 2016^5^; Goderis et al, 2014^26^
S/P	0.010 (±20%)	Gantt et al, 2016^5^; Goderis et al, 2014^26^
Asymptomatic newborns with hearing loss at birth		
Treatment		
Improvement of hearing loss	0.213 (±20%)^a^	Pasternak et al, 2018^33^
Progress of hearing loss	0.005 (±20%)^a^	Pasternak et al, 2018^33^
No treatment		
Improvement of hearing loss	0.033 (±20%)	Royackers et al, 2013^27^
Progress of hearing loss	0.033 (±20%)	Royackers et al, 2013^27^
Asymptomatic newborns without hearing loss at birth		
Late-onset hearing loss (no treatment)		
M/M	0.016 (±20%)	Salomè et al, 2020^25^; Goderis et al, 2014^26^
S/P	0.002 (±20%)	Salomè et al,2020^25^; Goderis et al, 2014^26^
Cost estimates, USD		
CMV PCR test	15 (7.5-37.5)	Estimated
Hearing check	37.5 (30-45)	Qiu et al, 2016^28^
Antiviral therapy	675 (600-1350)	Estimated
Hearing loss (annual)		
M/M^b^	300 (225-375)	Estimated
S/P	450 (375-525)	Qiu et al, 2016^28^
Cochlear implant (first y)^c^	30 000 (22 500-45 000)	Qiu et al, 2016^28^
Post-cochlear implant (annual)^d^	1500 (750-2250)	Qiu et al, 2016^28^
Health state QoL weights		
Hearing loss		
M/M	0.8 (0.78-0.82)	Cheng et al, 1999^34^; Crowson et al, 2017^35^
S/P	0.54 (0.52-0.56)	Cheng et al, 1999^34^; Crowson et al, 2017^35^
Post–cochlear implant	0.8 (0.78-0.82)	Cheng et al, 1999^34^; Crowson et al, 2017^35^
Cochlear implant ratio	0.5 (0.2-1.0)	Gantt et al, 2016^5^

Abbreviations: CMV, cytomegalovirus; M/M, mild to moderate; PCR, polymerase chain reaction; QoL, quality of life; S/P, severe to profound.

^a^ We assumed the effect of antiviral treatment lasted 6 years and considered the late-onset hearing loss would occur before age 6 years in the model.

^b^ We assumed that the annual cost of M/M hearing loss was less than S/P hearing loss, including the difference of hearing aid models and the cost of maintenance.

^c^ First-year costs of cochlear implant included the fixed cost of the initial cochlear implant system (implant and sound processor), preoperative assessments, including imaging and vestibular tests, and the hospital episode.

^d^ Cost includes cochlear implant spare parts, battery, maintenance, and replacement of sound processors.

Most of the health utility values were based on previous studies, including a meta-analysis of the cost utility of the cochlear implant.^34,35^ We assumed cochlear implant could restore health to at least M/M hearing loss. Therefore, we assigned the same health utility value for M/M hearing loss and the post–cochlear implant in our base model.

### Statistical Analysis

A 76-year cycle with a 1-year interval for the Markov model was specified according to life expectancy in China, with each cohort experiencing the currently observed age-specific mortality rates. Our model simulated the lifetime clinical course of the 3 strategies and projected expected average quality-adjusted life-years (QALYs) and costs for the base case cohort. We then calculated the incremental cost-effectiveness ratio (ICER) for 3 screening strategies. According to the World Health Organization (WHO) guidelines,^38^ an ICER that falls within 1 to 3 times the national gross domestic product (GDP) per capita ($9900 in 2018) is considered to be cost effective. The QALYs and costs were discounted at a 3.5% rate. Our analysis was conducted from the perspective of the Chinese health care system and included only direct medical costs. The number needed to treat was calculated as the reciprocal of the outcome event rate difference between targeted or universal screening and no screening for the same population.

### Sensitivity Analysis

A univariate sensitivity analysis was conducted to assess the effect of uncertainty in the prevalence of cCMVi, diagnosis and treatment costs, status transition probabilities, utilities, and cochlear implant ratio. The 95% CIs from a previous study were used as the upper and lower bounds of the health state utilities.^34,35^ A change of 20% of the original values was used for the transitional probabilities. Ranges of other parameters were assumed depending on the uncertainty of the base-case values. As data on the probability distributions of most of the parameter values were lacking, we decided not to perform a probabilistic sensitivity analysis (PSA). Analyses were conducted using TreeAge (TreeAge, LLC).

## Results

### Base Case Analysis

In the simulated synthetic cohort of Chinese live births, the prevalence of cCMVi was estimated to be 0.7% in the whole population. Based on the prevalence and the current practice in China (no screening strategy), 3675 (95% CI, 3559-3794) newborns (25% of symptomatic cases; Figure 1) received a diagnosis of cCMVi and antiviral therapy. In the targeted screening strategy, 225 000 newborns who failed a hearing screening underwent PCR tests for CMV. Among the total cases needing treatment, 41% of cases were treated with antiviral therapy, preventing 323 (95% CI, 289-360) cases of M/M hearing loss and 497 (95% CI, 454-543) cases of S/P hearing loss. For the universal screening strategy, which identified all the 105 000 newborns with infection, 15 783 cases (95% CI, 15 557-16 011) with cCMVi symptoms and/or hearing loss received antiviral treatment, leading to 1331 (95% CI, 1261-1404) cases of M/M hearing loss and 985 (95% CI, 925-1048) cases of S/P hearing loss prevented (Table 2). These data correspond with the numbers needed to treat of approximately 13 and 16 to prevent 1 case of CMV-related S/P hearing loss for targeted and universal screening strategies, respectively.

**Table 2.** Relative Performance of 3 cCMVi Screening Strategies

Characteristic	No. (95% CI)		
	No screening	Targeted screening	Universal screening
Newborns screened	0	225 000	15 000 000
cCMVi cases identified^a^	3675 (3559-3794)	7499 (7336-7664)	105 000 (104 369-105 635)
Cases receiving antiviral therapy	3675 (3559-3794)	6507 (6355-6662)	15 783 (15 557-16 011)
M/M			
Cases of hearing loss	9688 (9505-9873)	9365 (9185-9548)	8357 (8186-8531)
Hearing loss cases prevented	NA	323 (289-360)	1331 (1261-1404)
S/P			
Cases of hearing loss	3853 (3734-3974)	3356 (3245-3470)	2868 (2765-2973)
Hearing loss cases prevented	NA	497 (454-543)	985 (925-1048)

Abbreviations: cCMVi, congenital cytomegalovirus infection; M/M, mild to moderate; NA, not applicable; S/P, severe to profound.

^a^ It was assumed that 25% of symptomatic cases would have been clinically diagnosed without the screening.

Compared with no screening, targeted screening strategy increased QALYs by 38 415 and totals costs by $3 034 380. Universal screening increased QALYs by 126 540 and increased total costs by $264 114 150 compared with no screening. The ICER of targeted screening vs no screening was $79 per QALY gained. Compared with no screening, the incremental cost per QALY gained was $2087 for universal screening (Table 3). Based on WHO’s guidelines, targeted and universal screening strategies were cost-effective, with ICERs lying below 1 to 3 times China's GDP/capita.

**Table 3.** Cost-effectiveness Analysis of 3 Screening Strategies Applied to 15 Million Newborns

Strategy	Cost, USD	Effectiveness, QALY	Incremental cost, USD	Incremental effectiveness, QALY	ICER (USD/QALY)
**Cost and effectiveness undiscounted (0%)**					
No screening	777 176 181	1 093 156 905	NA		
Targeted screening	759 290 428	1 093 175 274	−17 885 752	18 370	−974 (dominant)
Universal screening	982 895 529	1 093 204 020	205 719 348	47 116	4366
Targeted screening	759 290 428	1 093 175 274	NA		
Universal screening	982 895 529	1 093 204 020	223 605 101	28 746	7779
**Cost and effectiveness discounted (3.5%)**					
No screening	276 374 232	1 092 623 960	NA		
Targeted screening	279 408 612	1 092 662 375	3 034 380	38 415	79
Universal screening	540 488 382	1 092 750 501	264 114 151	126 540	2087
Targeted screening	279 408 612	1 092 662 375	NA		
Universal screening	540 488 382	1 092 750 501	261 079 770	88 125	2963
**Cost and effectiveness discounted (5%)**					
No screening	203 943 553	1 092 547 158	NA		
Targeted screening	209 961 831	1 092 588 498	6 018 277	41 340	146
Universal screening	476 414 416	1 092 685 156	272 470 863	137 998	1974
Targeted screening	209 961 831	1 092 588 498	NA		
Universal screening	476 414 416	1 092 685 156	266 452 586	96 658	2757

Abbreviations: ICER, incremental cost-effectiveness ratio; NA, not applicable; QALY, quality-adjusted life-year.

### One-Way Sensitivity Analysis

**Figure 2.**
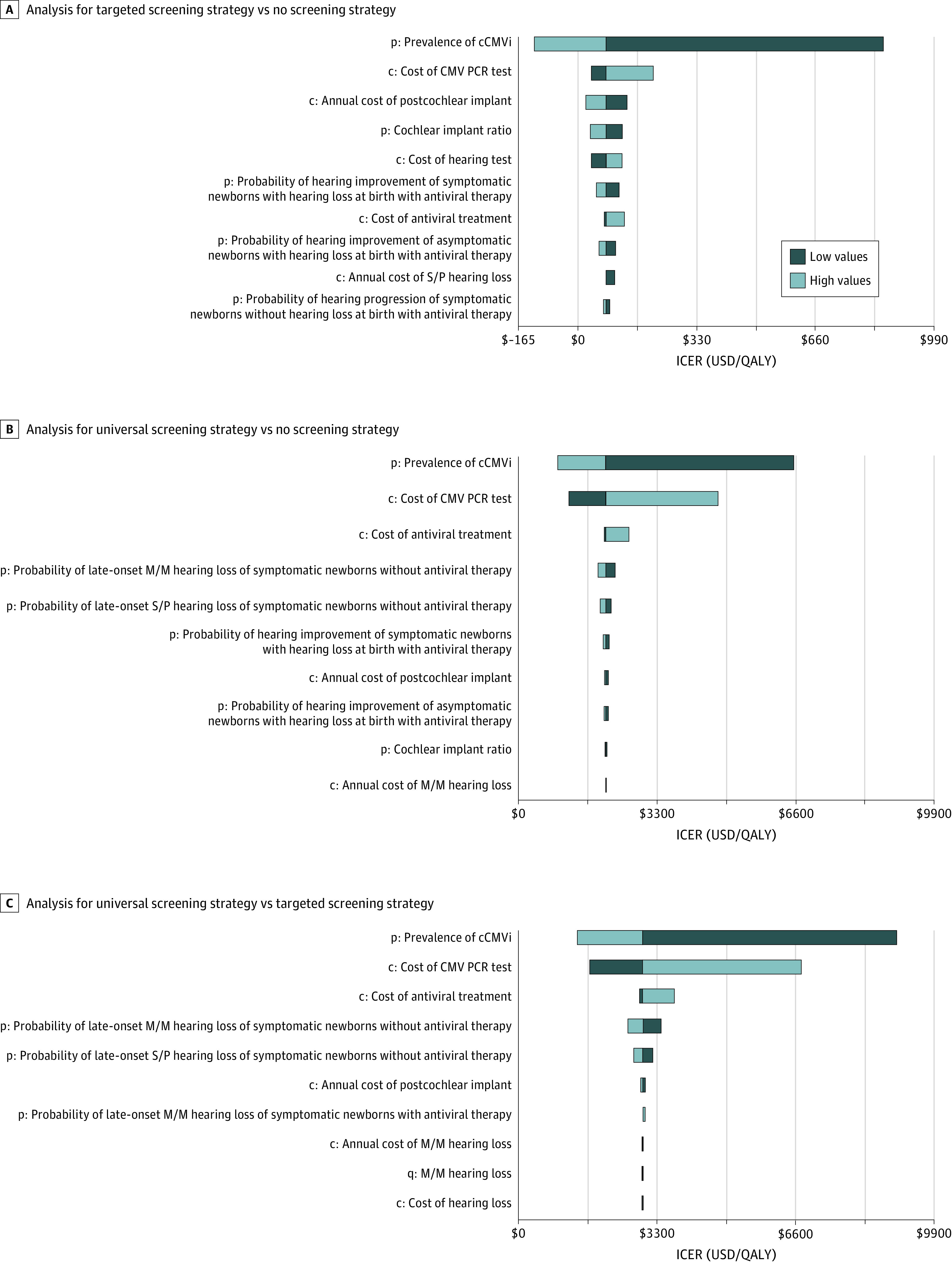
Tornado Diagrams for 1-Way Sensitivity Analysis of Incremental Cost-effectiveness Ratios (ICERs) A, Analysis for targeted screening strategy vs no screening strategy. B, Analysis for universal screening strategy vs no screening strategy. C, Analysis for universal screening strategy vs targeted screening strategy. c indicates cost; cCMVi, congenital cytomegalovirus infection; M/M, mild to moderate; p, probability; PCR, polymerase chain reaction; q, quality-of-life utility; QALY, quality-adjusted life-year; S/P, severe to profound; USD, US dollar.

The sensitivity analysis suggested that the variables that had a significant association with the cost-effectiveness of the screening strategies were the prevalence of cCMVi, cost of the PCR test, and cost of antiviral treatment (Figure 2; eTable in the Supplement). Compared with no screening, the ICERs of targeted screening and universal screening strategies were most sensitive to the prevalence of cCMVi, from $6532 per QALY gained for universal screening at prevalence of 0.2% to cost-saving for targeted screening at a prevalence of 2%. When prevalence was greater than 0.9%, targeted screening became cost-saving compared with no screening (eFigure 2 in the Supplement). Besides the prevalence of cCMVi, the ICER of universal screening was sensitive to CMV diagnosis and antiviral treatment costs. The ICER of universal screening compared with no screening fell to $1198 per QALY gained when the cost of a PCR test was $7.50. Compared with targeted screening, the ICER of universal screening increased to $3717 per QALY gained at VGCV treatment cost of $1350 (estimated based on the list price of Valcyte [Genentech]) (eTable and eFigure 2 in the Supplement).

## Discussion

Congenital cytomegalovirus is an invisible elephant in the room.^39^ Newborn screening provides an opportunity for presymptomatic identification and early intervention to prevent or mitigate the morbidity of cCMVi. Gantt et al^5^ suggested that both approaches could be cost effective or cost saving to the highly resourced health care system in the US. However, their approach might not be applicable to resource-limited areas. National or provincial newborn screening programs will need the cost-effectiveness evaluation for their own populations. Beginning in 1981, newborn screening started in Shanghai, and was followed by Beijing 8 years later. Currently, almost all provinces of China have launched newborn screening programs with congenital hypothyroidism, phenylketonuria, and hearing.^40^ The number of children with CMV-related disabilities is even greater than the number with better-known conditions, such as Down syndrome or congenital hypothyroidism. In contrast, little has been done to stop CMV from affecting newborns in China.

To our knowledge, this is the first study to estimate the costs and health effects of cCMVi screening program in China. Based on our analysis, compared with the current no screening practice, targeted and universal screening strategies would be highly cost-effective in reducing CMV-related disease burden based on the threshold recommended by WHO guidelines.^38^ The targeted screening strategy was cost-effective when compared with no screening (ICER = $79/QALY). Meanwhile, it was less costly but also less effective than the universal screening strategy, identifying 7% of all CMV infections and 41% of cases needing antiviral treatment. Given the current CMV prevalence in China, universal screening was found to be the most effective and cost-effective strategy among the screening strategies. Furthermore, the literature has estimated that the lifetime indirect cost (ie, productivity losses) associated with hearing loss could be around 2 to 5 times the amount of direct costs.^41^ Newborn screening and early intervention for children with CMV-related hearing loss may be more cost-effective given the expected gains in lifetime earnings. Given that universal and targeted screening are cost-effective compared with no screening, policy makers need to decide which strategy to adopt between universal and targeted screening. Whether to roll out universal screening or first implement targeted screening before scaling it to universal screening could depend on the geosocioeconomic gap among the Chinese provinces. This is necessary, given that there is a substantial regional diversity in China regarding the coverage of PCR laboratories and cochlear implant surgery.

There are several strengths to this evaluation. The evaluation estimated short-term indicators, including the number of newborns with cCMVi or the infants receiving antiviral therapy, as well as long-term outcomes, such as QALY gains. Cost variables used in the model were all based on Chinese studies, including a recent observational study of pediatric cochlear implant.^28^ Country-specific cCMVi management and cost data could lead to a realistic assessment for the Chinese setting. In addition, the uncertainty surrounding the values of the model parameters was incorporated and assessed using a sensitivity analysis.

On one hand, children with cCMVi treated with GCV intravenously for 6 weeks may still experience hearing loss. On the other hand, more asymptomatic infants would be discovered with a universal screening strategy, while antiviral therapy for asymptomatic infants is still not recommended.^10^ Compared with GCV, VGCV has superior bioavailability and better adherence with oral treatment, even for 6 months.^42^ A phase II trial of oral VGCV therapy in infants with asymptomatic cCMVi without SNHL currently is under way (ClinicalTrials.gov identifier, NCT03301415). Moreover, as 10% of asymptomatic infants without hearing loss are at risk of developing late-onset sequelae,^25^ the predictive markers to identify those at high risk of developing sequelae are quite desirable.

Cochlear implant coverage may have a critical association with the ICER because of the high cost (device cost and device-related expenses) and averted disability. As almost all children with S/P deafness receive at least 1 implant in high-income countries, the option of no implant was not considered. However, the number of cochlear implant patients pales compared with the much larger population of patients with deafness who had no access to the surgery in China.^28,43^ This emphasizes a need to tailor hearing-related health services in middle-income countries to the newborn screening programs.

### Limitations

First, we only estimated the association of cCMVi screening with hearing health. If other CMV-related disabilities, such as cognitive deficit and vision impairment, were considered, effectiveness of cCMVi screening might increase significantly.^41^ Second, the efficacy and safety of antiviral therapy has not been well defined.^32^ The conceptual transition model was constructed by a team of clinical experts in cCMVi based on limited literature. Third, our model was based mainly on one previously developed in Canada^5^ and the input parameter set (eg, transition probabilities) on data from European countries.^24,25,26,27^ Because of a lack of adequate epidemiological data in China for calibration, it is uncertain whether our model accurately fit the Chinese setting. Fourth, it was not possible to conduct a PSA because of data limitations regarding the probability distributions of most of the parameter values. Fifth, as there is no official willingness-to-pay threshold value in China, it cannot be concluded with certainty whether the base case results are perceived as cost-effective. Ochalek et al^44^ suggested that the cost-effectiveness threshold that reflected health opportunity costs may be below 1 × GDP per capita in China. Finally, given the lack of data on societal factors associated with health care costs (eg, sex, age, education, and level of dependency), additional investigation on the broader societal effect of different cCMVi screening strategies is warranted.

## Conclusions

This evaluation demonstrated that universal screening could be cost-saving and more effective compared with targeted screening or no screening. Many children with cCMVi in China could benefit each year from newborn CMV screening, early detection, and interventions. The results presented in this study could be used by Chinese policy makers to make an informed decision about the scale-up of universal screening programs. While the results are specific to China, the model may be easily adapted to health settings in other middle-income countries. Further research is warranted to include long-term indirect costs, estimate health state utilities in the Chinese population, and conduct PSAs to reflect uncertainty in the economic estimates.

## Notes

Supplement. **eTable.** Influential variables from one-way sensitivity analysis **eFigure 1.** State-transition diagrams for the progression of cytomegalovirus (CMV)-related hearing loss **eFigure 2. **One-way sensitivity analysis on the incremental cost-effectiveness ratios (ICERs)
