# Supplementary material for: Estimated Cost-effectiveness of Newborn Screening for Congenital Cytomegalovirus Infection in China Using a Markov Model
Source: JAMA Netw Open. 2020 Dec 4;3(12):e2023949. doi: 10.1001/jamanetworkopen.2020.23949 (PMC7718603; doi:10.1001/jamanetworkopen.2020.23949)
Supplement: Supplement. — eTable. Influential variables from one-way sensitivity analysis eFigure 1. State-transition diagrams for the progression of cytomegalovirus (CMV)-related hearing loss eFigure 2. One-way sensitivity analysis on the incremental cost-effectiveness ratios (ICERs) [file jamanetwopen-e2023949-s001.pdf]

Supplemental Online Content

Chen K, Zhong Y, Gu Y, et al. Estimated cost-effectiveness of newborn screening for congenital cytomegalovirus infection in China using a Markov model. *JAMA Netw Open*. 2020;3(12):e2023949. doi:10.1001/ jamanetworkopen.2020.23949

eTable. Influential variables from one-way sensitivity analysis

eFigure 1. State-transition diagrams for the progression of cytomegalovirus (CMV)-related hearing loss

eFigure 2. One-way sensitivity analysis on the incremental cost-effectiveness ratios (ICERs)

© 2020 Chen et al. *JAMA Network Open.*

eTable. Influential variables from one-way sensitivity analysis

Influential variable	Range	ICER Range (USD/QALY)[b] Targeted screening vs. No screening	Universal screening vs. No screening	Universal screening vs. Targeted screening
Prevalence of cCMVi	0.002-0.02	848-Cost saving	6532-932	9011-1390
Cost of CMV PCR test (CNY)	7.5-37.5	35-210	1199-4754	1705-6734
Cost of antiviral treatment (CNY)	600-1350	74-129	2027-2628	2879-3717
Annual cost of Post-CI (CNY)	750-2250	135-23	2239-2132	3211-3107

Abbreviations: cCMVi, congenital cytomegalovirus infection; CI, cochlear implant; ICER, incremental cost-effectiveness ratio; PCR, polymerase chain reaction.

[a]The influential variables were determined by ranking the ICER range in the one-way sensitivity analyses for the model variable input.

[b]Discounted at 3.5% annual rate.

© 2020 Chen et al. *JAMA Network Open.*

eFigure 1. State-transition diagrams for the progression of cytomegalovirus (CMV)-related hearing loss

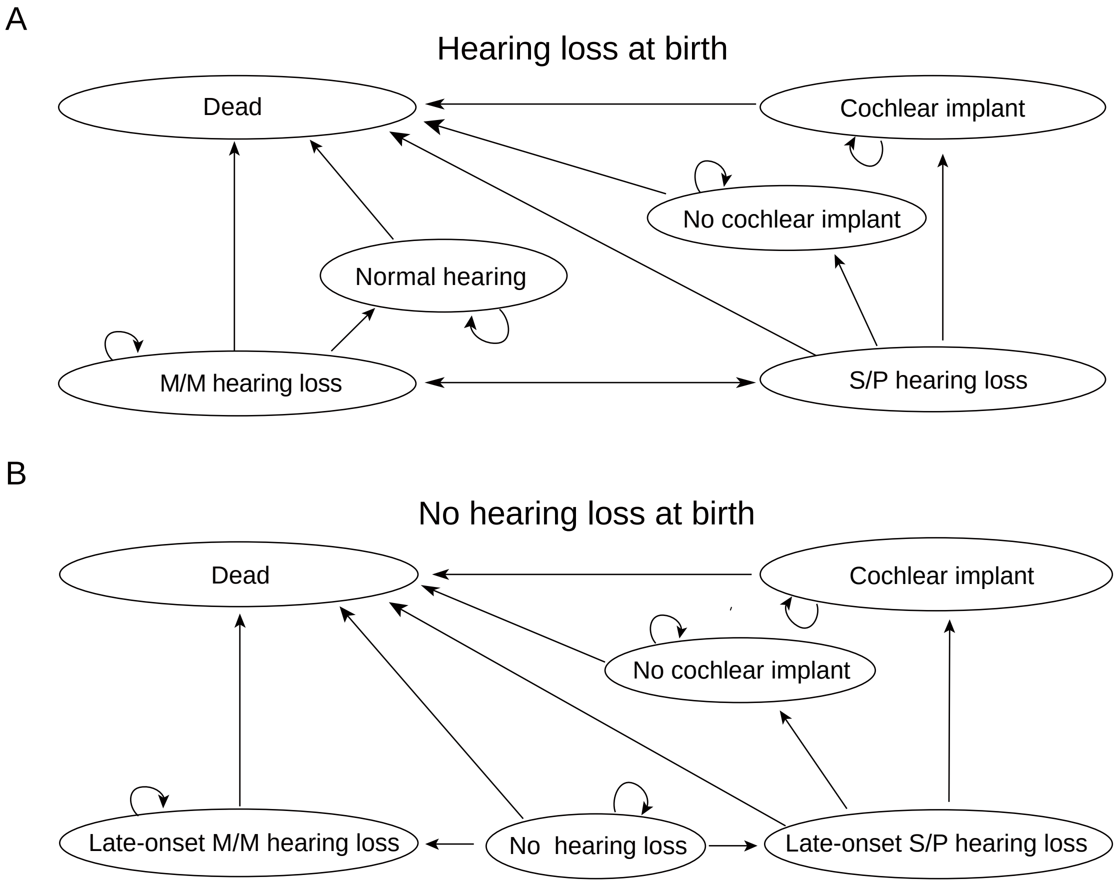

Abbreviations: M/M, mild to moderate; S/P, severe to profound.

© 2020 Chen et al. *JAMA Network Open.*

eFigure 2. One-way sensitivity analysis on the incremental cost-effectiveness ratios (ICERs)

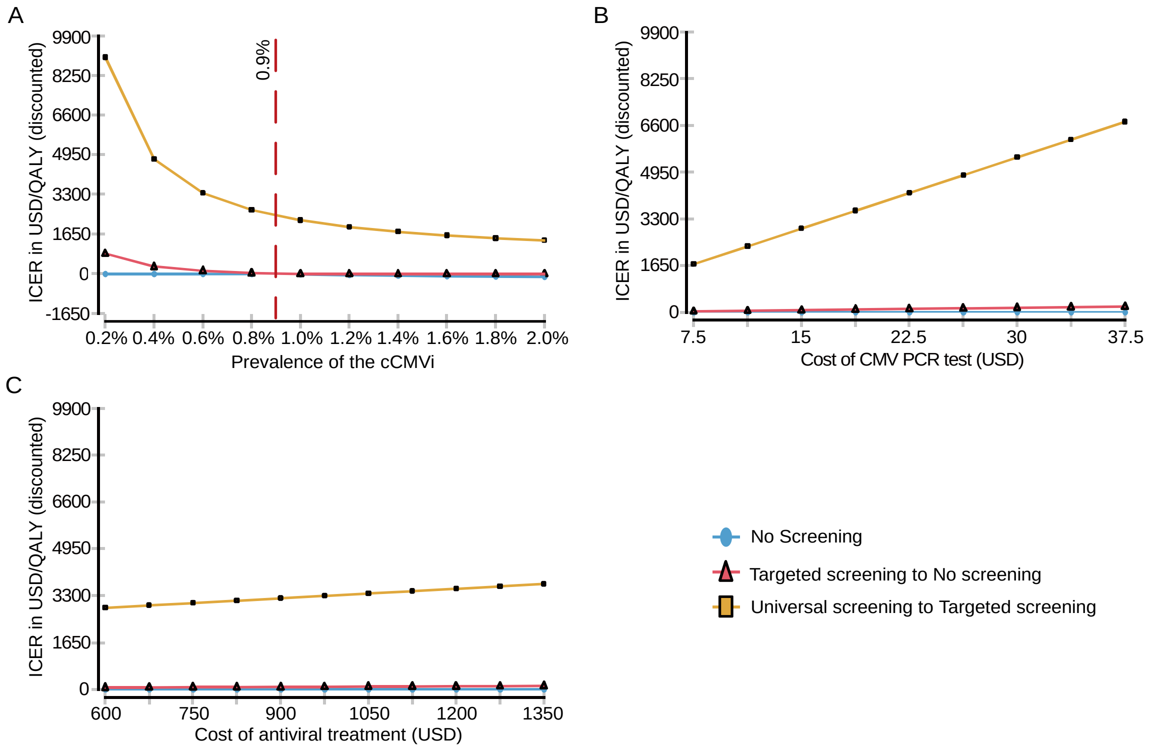

(A) Analysis for the impact of the prevalence of the cCMV on ICER. (B) Analysis for the impact of the cost of CMV PCR test on ICER. (C) Analysis for the impact of the cost of antiviral treatment on ICER.

Abbreviations: cCMVi, congenital cytomegalovirus infection; PCR, polymerase chain reaction; QALY, quality-adjusted life year.

© 2020 Chen et al. *JAMA Network Open.*
